# Hyperosmolarity potentiates toxic effects of benzalkonium chloride on conjunctival epithelial cells in vitro

**Published:** 2012-04-06

**Authors:** Chloé Clouzeau, David Godefroy, Luisa Riancho, William Rostène, Christophe Baudouin, Françoise Brignole-Baudouin

**Affiliations:** 1 INSERM, U968, Paris, France; 2 UPMC Univ Paris 06, UMR_S 968, Institut de la Vision, Paris, France; 3 CNRS, UMR_7210, Paris, France; 4 Centre Hospitalier National d’Ophtalmologie des Quinze-Vingts, Paris, France; 5 Hôpital Ambroise Paré, Assistance Publique - Hôpitaux de Paris; Université Versailles Saint-Quentin-en-Yvelines, France; 6 Université Paris Descartes, Faculté des Sciences Pharmaceutiques et Biologiques, Laboratoire de Toxicologie, Paris, France

## Abstract

**Purpose:**

Benzalkonium chloride (BAK), the most commonly used preservative in eye drops, is known to induce ocular irritation symptoms and dry eye in long-term treated patients and animal models. As tear film hyperosmolarity is diagnostic of some types of dry eye disease, we determined in vitro on conjunctival epithelial cells the cytoxicity of BAK in hyperosmolar conditions through cell viability, apoptosis, and oxidative stress assays.

**Methods:**

The Wong Kilbourne derivative of Chang conjunctival epithelial cells were cultured for 24 h or 48 h either in NaCl-induced hyperosmolar conditions (400–425–500 mOsM), in low concentrations of BAK (10^−4^%, 3.10^−4^%, and 5.10^−4^%), or in combination of both. We investigated cell viability through lysosomal integrity evaluation, cell death (cell membrane permeability and chromatin condensation), and oxidative stress (reactive oxygen species, superoxide anion) using spectrofluorimetry. Immunohistochemistry was performed for cytoskeleton shrinkage (phalloidin staining), mitochondrial permeability transition pore (cytochrome c release), the apoptosis effector active caspase-3, and the caspase-independent apoptosis factor AIF. We also observed early effects induced by the experimental conditions on the conjunctival cell layers using phase contrast imaging of live cells.

**Results:**

As compared to standard culture solutions, hyperosmolar stress potentiated BAK cytotoxicity on conjunctival cells through the induction of oxidative stress; reduction of cell viability; cell membrane permeability increase; cell shrinkage with cell blebbing, as shown in phase contrast imaging of live cells; and chromatin condensation. Like BAK, but to a much lesser extent, hyperosmolarity increased cell death in a concentration-dependent manner through a caspase-dependent apoptosis characterized by a release of cytochrome c in the cytoplasm from mitochondria and the activation of caspase-3. Moreover, the caspase-independent apoptosis factor AIF was found translocated from mitochondria to the nucleus in both conditions.

**Conclusions:**

This study showed increased cytotoxic effects of BAK in hyperosmotic conditions, with characteristic cell death processes, namely caspase-dependent and independent apoptosis and oxidative stress. As BAK is known to disrupt tear film, which could promote evaporative dry eye and tear hyperosmolarity, BAK could promote the conditions enhancing its own cytotoxicity. This in vitro hyperosmolarity model thus highlights the risk of inducing a vicious cycle and the importance of avoiding BAK in patients with dry eye conditions.

## Introduction

The ocular surface is the most environmentally exposed mucosal surface of the body, protected only by the lids and tear film. The production and turnover of the tear functional unit are essential for maintaining the ocular surface in good health and efficiently preventing foreign particles, pathogens, allergens, or irritants from entering or injuring the eye. Dry eye is a multifactorial worldwide syndrome affecting one million people, with a prevalence in the general population over 50 years of 3%–15% [1]. Dry eye is characterized by eye irritation symptoms, blurred and fluctuating vision, tear film instability, increased tear osmolarity, and impairment of ocular surface epithelia [2-4]. Dry eye symptoms result from a poorly lubricated ocular surface inducing inflammation and cell apoptosis. Tear hyperosmolarity and inflammation of the ocular surface epithelium are now considered the core mechanisms underlying dry eye disease and causing a vicious cycle in which an abnormal tear film stimulates a series of biologic events that further impair tear function [1,5]. In these conditions, the ocular surface’s ability to maintain tear film integrity and respond to environmental (indoor and outdoor pollutants, dust particles, pollens) and iatrogenic (topical ocular drugs and preservatives) challenges is impaired.

To understand the mechanisms leading to or induced by dry eye disease, many experimental models have been developed in animals and cell lines. Mice, rats, rabbits, and even dogs have been investigated [6-15]. Primary cultures of corneal epithelial cells [16,17] or limbal cells [18] and corneal cell lines [19,20] have been extensively studied in hyperosmolarity conditions to evaluate apoptosis and signaling pathways [18-23]. Hyperosmolarity was shown to induce apoptosis in vitro on corneal or limbal cells through mitochondrial depolarization, cytochrome c release, and an increase in caspase 3/7 and 9 activity, confirming a caspase-dependent mechanism [24,25]. Little is known, however, about conjunctival epithelium behavior in hyperosmolar conditions, even if a few studies have investigated the ocular surface tissues more extensively.

Xiong et al. [26] and Lin et al. [27] successfully developed in vivo dry eye models in rabbits and mice, respectively, using topical instillations of benzalkonium chloride (BAK). BAK is the most commonly used preservative in eyedrops and is known for its toxic and proapoptotic effects on the ocular surface [28-31]. BAK is a quaternary ammonium presenting tensioactive properties that may disrupt the lipid layer of the tear film, thus increasing tear evaporation and causing tear film instability [32]. Additionally, it was shown to destroy goblet cells [33,34] and may directly play a role in iatrogenic dry eye development. Moreover, BAK was widely suspected of being the most important factor causing dry eye in glaucoma patients treated over a long-term [35-42], a concept that is now clearly supported by the use of BAK as an inducer of dry eye in experimental models. These findings directly demonstrate the interactions between tear film, the ocular surface, and BAK, through possible interactions with hyperosmotic stress and inflammatory conditions, and thus show that BAK is a reliable dry eye model.

The aim of the present study was to investigate the relationships between hyperosmolarity and BAK in vitro through their respective and combined proapoptotic and pro-oxidative effects on conjunctival epithelial cells, to determine whether BAK toxicity would be enhanced in hyperosmotic conditions, mimicking a frequent clinical condition in which dry eye patients receive artificial tears preserved with a chemical compound that may both promote and be potentiated by tear hyperosmolarity.

## Methods

### Cell line

The Wong Kilbourne derivative of Chang conjunctival epithelial cell line (WKD; clone 1–5c-4, American Type Culture Collection [ATCC, Manassas, VA] certified cell line [CCL], 20.2) was cultured under standard conditions (moist atmosphere, 5% CO_2_, 37 °C) in Dulbecco minimum essential medium (DMEM, 340 mOsM±20 mOsM) supplemented with 10% fetal bovine serum (FBS), 1% glutamine (200 mM stock solution), and 1% penicillin (10,000 units/ml) and streptomycin (10,000 µg/ml) for 24h to reach confluence before challenges. This cell line has been used previously for toxicological in vitro studies despite the presence of a small amount of HeLa cells. A toxicological study aimed at comparing Wong Kilbourne cell line and IOBA conjunctival cell line confirmed that the two cell lines presented the same toxic responses [28,29,43].

All reagents were purchased from Gibco (Cergy Pontoise, France).

### Cell challenge conditions

At confluence, cells were grown for 24 or 48 h: (1) in hyperosmolar media defined by different osmolarities, assessed using an osmometer (Roebling 13DR, Berlin, Germany), in accordance with previous studies [17,18,20], ranging from standard conditions at 340 (iso-osmolar culture medium), to 430, 450, or 500 mOsM, achieved by adding 50, 60, or 90 mM sodium chloride (NaCl, Fluka, Buchs, Switzerland) in supplemented DMEM medium, called HO50 mM, HO60 mM, and HO90 mM, respectively; (2) in media containing BAK (Fluka) at 10^−4^%, 3.10^−4^%, or 5.10^−4^% (345 mOsM±5 mOsM); or (3) in a combination of hyperosmolarity and BAK, by crossing the three osmotic stresses with the three BAK concentrations.

### Phase contrast imaging of live cells

Cells were cultured for 24 h on 0.17-mm-diameter Delta TPG uncoated culture dishes (O420041500C; Bioptechs, Butler, NJ), then monitored at the microscope stage (Eclipse 80i; Nikon, Tokyo, Japan) every 5 s with a charge-coupled device (CCD) camera (ORCA-03G; Hamamatsu, Hamamatsu, Japan). Videos highlighted morphological changes of cell-size decrease, membrane blebbing, formation of apoptotic bodies, and cell detachment. Cells were first observed in 1 ml DMEM before being exposed for 96 s to the most challenging stresses, HO90 mM, BAK10^−2^%, and a combination of HO90 mM and BAK10^−2^%, by adding 100 µl of HO900 mM, BAK10^−1^%, and HO900 mM associated with BAK10^−1^% solutions.

### Spectrofluorimetric cytotoxicity assays

Cells were first seeded at 10^5^ cells/ml in 96-well microplates (Corning-Costar, Corning, NY) for 24 h incubation to reach confluence in standard medium before cell challenges for 24–48 h in hyperosmolar or toxic conditions. The neutral red uptake (NRU), YO-PRO-1, Hoechst 33342, 2,7-dichlorodihydrofluorescein diacetate (H2DCF-DA), and dihydroethidine (HE) assay methods were previously described and will be briefly explained [28]. Fluorescence was measured using a microplate fluorometer (Infinite M1000; Tecan, Lyon, France).

### NRU/cell viability (CV) assay

Viable cells incorporate NR (Fluka), a cationic dye that diffuses through the cell membrane to bind the lysosomal matrix. This lysosomal membrane integrity is correlated with CV. After challenges, cells were incubated in 200 µl of 50 µg/ml NR in DMEM for 3 h in standard conditions, washed in PBS (D-PBS 1×; Gibco), incubated for 15 min in a lysis solution (1% acetic acid/50% ethanol/49% H_2_O) under agitation, and then fluorescence was measured (excitation, 535 nm; emission, 600 nm).

### Plasma-membrane permeability (PMP)/apoptosis evaluation

YO-PRO-1 (Fluka) binds DNA after opening specific membrane pore P2X7 (purinergic receptor) activated during early apoptosis through extracellular ATP (ATP) [44], making it a cell membrane permeabilization marker. After challenges, 200 µl/well of a 2 µM (1.26 mg/ml) YOPRO-1 solution in PBS was added for 10 min at room temperature (RT), in the dark before fluorometry analysis (excitation, 491 nm; emission, 509 nm).

### Caspase-3 activation assessment/apoptosis evaluation

Caspase-3 activation was detected using the Fluorometric Assay Kit following the manufacturer’s instructions (Biovision, Mountain View, CA). Absorbance from cells with and without 24 h treatment was compared to determine relative caspase activation.

### Chromatin condensation assessment/apoptosis evaluation

Hoechst 33342 (Invitrogen) is a UV fluorescent probe used to evaluate cell chromatin condensation entering both apoptotic and living cells. PI is used to discriminate necrotic cells since this dye competes with Hoechst for binding to DNA. After challenges, 200 µl of a solution containing Hoechst 33342 (0.5 µg/ml) and PI (0.05 µg/ml) in PBS was added for a 30 min incubation at RT, in the dark before fluorometry analysis (excitation, 360 nm; emission, 450 nm).

### Oxidative stress evaluation/H_2_DCF-DA and HE assays

Oxidative stress was assessed by reactive oxygen species (ROS) evaluation. Two different fluorometric methods were used: H2DCF-DA (Interchim, Montluçon, France) detects hydroperoxide in cells (excitation, 490 nm; emission, 535 nm), whereas HE (Interchim) detects superoxide anion in viable cells (excitation, 490 nm; emission, 600 nm) distinguishing O_2_**.^−^** from H_2_O_2_. After challenges, 200 µl of a 5 µM (1.58 µg/ml) HE solution in PBS or a 20 µM (9.75 µg/ml) H_2_DCF-DA solution was added for a 20 min incubation at 37 °C in moist atmosphere with 5% CO_2_ before fluorometry analysis.

### Apoptosis immunodetection

Caspase-dependent and -independent apoptosis was explored by highlighting specific markers (cytochrome c release, active caspase-3, and apoptosis-inducing factor, AIF, respectively). Cells were grown on round sterile cover glasses (diameter, 14 mm; Menzel GmbH, Germany), washed, fixed in 4% paraformaldehyde-PBS, and permeabilized in 0.3% Triton X-100 (Sigma-Aldrich, Saint-Quentin Fallavier, France). This was followed by a 1% BSA (BSA, Sigma-Aldrich) incubation for 30 min and a 1 h staining with the primary antibodies against AIF, cytochrome c (1:100; Sigma-Aldrich), and active caspase-3 (1:100; BDPharmingen, Franklin Lakes, NJ). The cells were then incubated for 1 with the secondary antibody Alexa Fluor^®^ 488 (1:500, goat anti-rabbit and anti-sheep; Molecular Probes, Invitrogen) before a 30 min incubation with phalloidin (1:40, Alexa Fluor^®^ 546; Invitrogen) and a 15 min incubation with DAPI (1:2,000; Sigma-Aldrich). Cover glasses were then mounted with Mountant PermaFluor^®^ (Thermo Fisher Scientific, Courtaboeuf, France) before observation with an epifluorescence microscope (Leica DM6000B, Rueil-Malmaison, France).

### Statistical analyses

All experiments were performed in triplicate, and the groups were compared using one-way ANOVA (ANOVA) followed by the Bonferroni adjustment (GraphPad, GraphPad Software, La Jolla, CA).

## Results

### Phase contrast video

**Figure 1:**
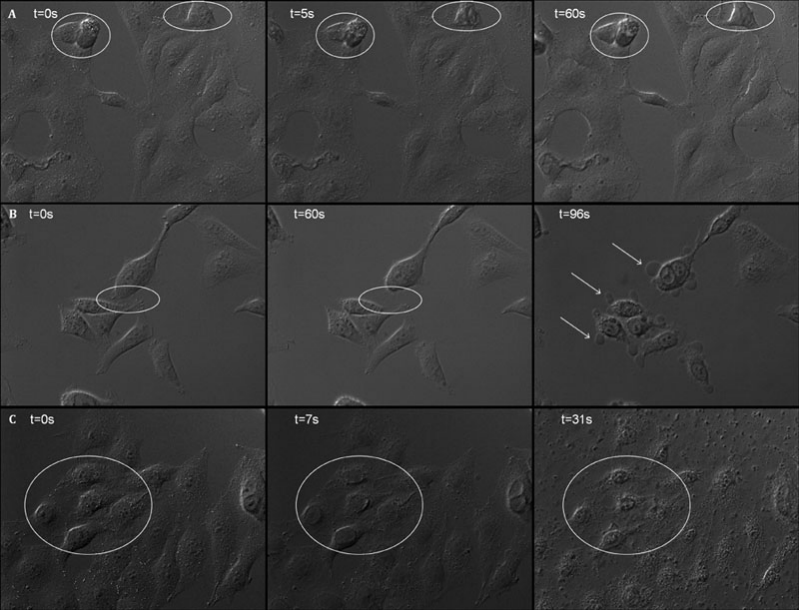
Phase contrast imaging of WKD live cells in DMEM (t=0 s) stressed at t=2 s by complementing media to obtain final concentration of HO90 mM (**A**; animation1), BAK10^−2^% (**B**: animation 2), and HO90 mM+BAK10^−2^% (**C**; animation3). First line: HO90 mM at t=0 s, t=5 s, and t=60 s; HO90 mM stress induced a cell-volume decrease for 3 s after stress observed at t=5 s before returning to almost normal size ( t=31 s), then a very discreet cytoplasmic retraction was observed. Second line: BAK10^−2^% at t=0 s, t=60 s, and t=96s ; BAK induced a progressive cytoplasm shrinkage observed from t=3 s to t=60 s (circles) and followed by intense cell blebbing (arrows, from t=60 s) before cell death. Last line: HO90 mM+BAK10^−2^% at t=0 s, t=7 s, and t=31 s. Cells exhibited profound alteration just after stress, such as disruption of plasma membrane and nuclear fragmentation leading to irreversible cell death already visible at t=7 s.

WKD cells exhibited morphological changes (Figure 1) with a cell-size decrease (Figure 1A), cell blebs (Figure 1B), and cell detachment (Figure 1C) after the different stresses. Under hyperosmolar conditions, cells exhibited a reversible cell-size decrease for 3 s after stress before returning to normal cell size (at t=20 s); no visible changes were observed until t=31 s, then a very discreet cytoplasmic retraction was observed. Under BAK, stress cells took longer to respond and showed irreversible morphological changes (cytoplasm retraction visible from t=2–60 s and cell blebs from t=60 s) leading to cell death at t=96 s, whereas imposing both stresses induced irreversible cell death at t=7 s.

### Spectrofluorimetric cytotoxicity assays

**Figure 2:**
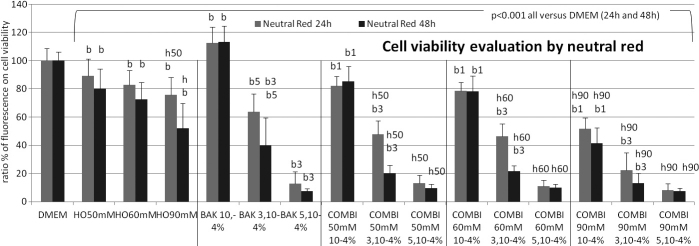
Cell viability (neutral red uptake assay) at 24 h (gray bars) and 48 h (black bars). The results were expressed as percentages (means±SD) of the 100% of control DMEM. When compared to DMEM, significant cytotoxic effects (p<0.001) were observed in HO conditions in an HO-dependent manner, and in BAK solutions in a BAK concentration- and time-dependent manner after 24 h and 48 h, except for the lowest concentration of BAK10−4%; the associations of both stresses in all experiments were more cytotoxic than their respective HO or BAK conditions. The following letter codes were used for statistical comparisons with (b) all BAK concentrations, (b1) BAK10−4%, (b3) BAK5.10−4%, (b5) BAK3.10−4%, (h) all HO solutions, (h50) HO50 mM, (h60) HO60 mM, (h90) HO90 mM, corresponding to a statistically significant difference at p<0.001.

**Figure 3:**
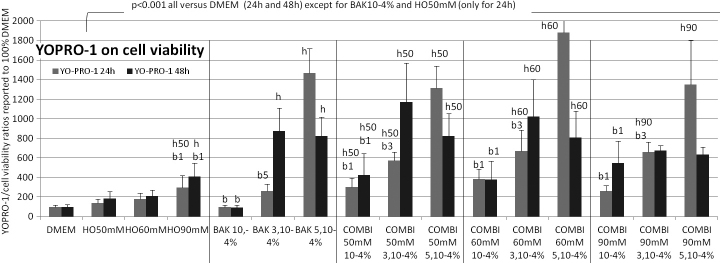
Plasma-membrane permeability (YO-PRO-1 assay) at 24 h (gray bars) and 48 h (black bars). The results expressed as percentages (means±SD) of the 100% of control DMEM and signals emitted by cell population are reported over the neutral red test as an indication of viable cells. At 24 h and 48 h, incubation in HO60 mM, HO90 mM, BAK3.10−4%,  BAK5.10−4% and combinations of BAK with all HO conditions induced an increase (p<0.001) in plasma-membrane permeability compared to control. Combinations of BAK10-4% with all HO increased detection level (p<0.001) compared to BAK10-4% alone. The following letter codes were used for statistical comparisons with (b) all BAK concentrations, (b1) BAK10−4%, (b3) BAK5.10−4%, (b5) BAK3.10−4%, (h) all HO solutions, (h50) HO50 mM, (h60) HO60 mM, (h90) HO90 mM, corresponding to a statistically significant difference at p<0.001.

**Figure 4:**
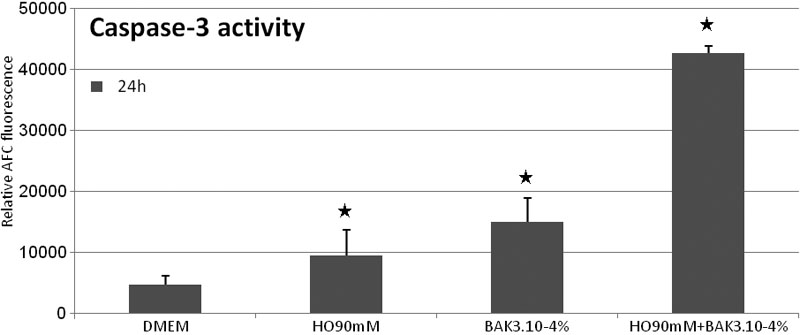
Capase-3 activity. HO90mM, BAK3.10^−4^%, and HO90 mM+BAK3.10^−4^% induced at 24 h an increase of caspase-3 activity with respective levels of 9458.3±1461.3, 15010.5±3947.8, and 42688.7+/−1142.7 compared to DMEM (4742.4±1406.2) (★ p<0.01). The combination of BAK3.10^−4^% with HO90 mM induced considerable caspase-3 activity compared to HO90 mM and BAK3.10^−4^% alone (p<0.01).

**Figure 5:**
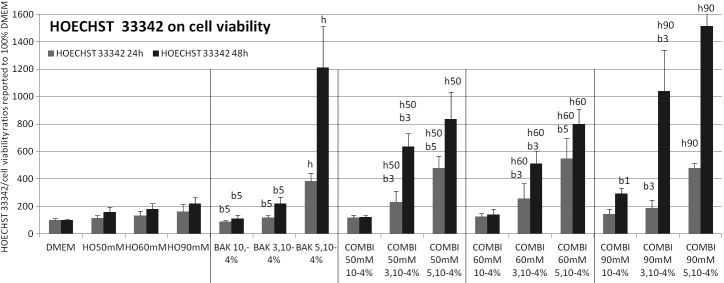
Chromatin condensation (Hoechst 33342 assay) at 24 h (gray bars) and 48 h (black bars). The results expressed as percentages (means±SD) of the 100% of control DMEM and signals emitted by cell population are reported over the neutral red test as an indication of viable cells. At 24 h and 48h, incubations with HO, BAK and all combinations induced an increase (p<0.001) in fluorescence levels compared to control (except at 24 h for HO50 mM, BAK10−4% and combinations of BAK10−4% with all HO). All combinations of BAK3.10−4% with HO were statistically different (p<0.001) compared to BAK3.10-4% alone. The following letter codes were used for statistical comparisons with (b) all BAK concentrations, (b1) BAK10−4%, (b3) BAK5.10−4%, (b5) BAK3.10−4%, (h) all HO solutions, (h50) HO50 mM, (h60) HO60 mM, (h90) HO90 mM, corresponding to a statistically significant difference at p<0.001.

**Figure 6:**
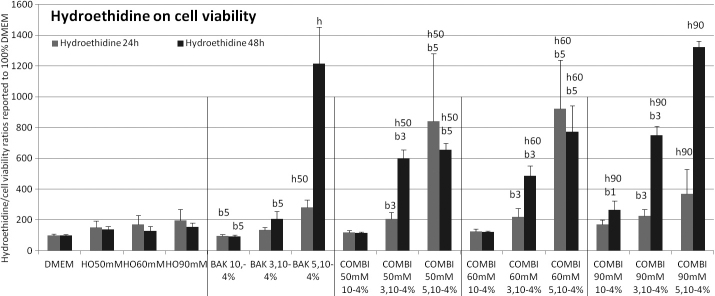
Oxidative stress evaluation/superoxide anion production (hydroethidine assay) at 24 h (gray bars) and 48 h (black bars). The results expressed as percentages (means±SD) of the 100% of control DMEM and signals emitted by cell population are reported over the neutral red test as an indication of viable cells. Compared to control, a significant (p<0.001) increase of fluorescence values at 24 h and 48 h was observed in HO and BAK conditions alone (except for HO50 mM and BAK3.10−4% at 24 h, and BAK10−4% at 24h and 48h). The combination of any HO with BAK3.10−4% or BAK5.10−4% induced a superoxide anion increase (p<0.001). The following letter codes were used for statistical comparisons with (b) all BAK concentrations, (b1) BAK10−4%, (b3) BAK5.10−4%, (b5) BAK3.10−4%, (h) all HO solutions, (h50) HO50 mM, (h60) HO60 mM, (h90) HO90 mM, corresponding to a statistically significant difference at p<0.001.

**Figure 7:**
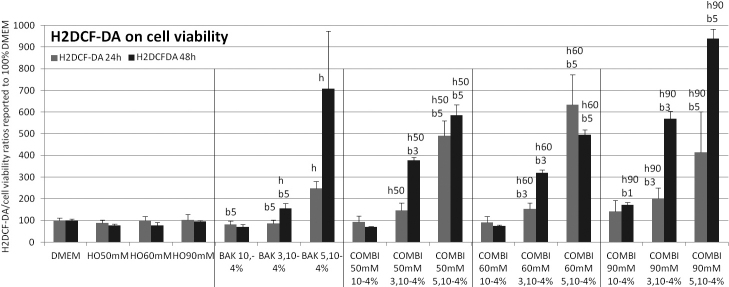
Oxidative stress evaluation/reactive oxygen species (COO^-^, ONOO^-^) and mainly hydrogen peroxide production (H_2_DCF-DA assay) at 24 h (gray bars) and 48 h (black bars). The results expressed as percentages (means±SD) of the 100% of control DMEM and signals emitted by cell population are reported over the neutral red test as an indication of viable cells. At 24 h and 48 h, compared to control, incubation in BAK5.10−4% and  combinations of any HO with BAK3.10−4% and BAK5.10−4% induced a superoxide anion increase (p<0.001), except for HO50 mM and HO60 mM associated with BAK3.10−4% at 24 h. The following letter codes were used for statistical comparisons with (b) all BAK concentrations, (b1) BAK10−4%, (b3) BAK5.10−4%, (b5) BAK3.10−4%, (h) all HO solutions, (h50) HO50 mM, (h60) HO60 mM, (h90) HO90 mM, corresponding to a statistically significant difference at p<0.001.

These assays are illustrated in Figure 2, Figure 3, Figure 4, Figure 5, Figure 6, and Figure 7 with histograms grouped in threes after the control DMEM normalized at 100%, to show the respective effects of (1) HO conditions, (2) BAK conditions, and combinations of: (3) HO50 mM with BAK10^−4^%, 3.10^−4^%, 5.10^−4^%; (4) HO60 mM with BAK10^−4^%, 3.10^−4^%, 5.10^−4^%; and (5) HO90 mM with BAK10^−4^%, 3.10^−4^%, 5.10^−4^%.

### Neutral red/cell viability assay (Figure 2)

Cell viability was assessed at 24 h and 48 h following different challenges, hyperosmolarity, BAK and the associations of both conditions.

At 24 h, compared to DMEM, incubations in HO, BAK, and combinations induced a CV decrease (p<0.001), except for BAK10^−4^%, which induced a slight CV increase (p<0.001). Incubation with BAK3.10^−4^% or BAK5.10^−4^% for 24 h decreased CV compared to DMEM (p<0.001). Whatever the concentration tested, BAK always decreased CV more than any HO condition alone. In HO associated with BAK, CV was further reduced compared to BAK alone, causing a significant cytotoxicity dependent on both HO and BAK concentrations. All BAK10^−4^% combinations were significantly more cytotoxic (p<0.001) than BAK10^−4^% alone. BAK3.10^−4^% combinations with HO50 mM, HO60 mM, or HO90 mM were significantly more cytotoxic (p<0.001) than BAK3.10^−4^% alone (p<0.001), but BAK5.10^−4^% combinations with all HO combinations did not differ from BAK5.10^−4^%, which was too cytotoxic for an additive effect to be observed.

At 48 h, with a similar cytotoxicity profile, incubation in the same conditions (HO, BAK, and combinations) further induced a time-dependent CV decrease compared to 24 h. All combinations induced additive effects (p<0.001) compared to BAK solutions except for BAK 5.10^−4^% combinations with HO60 mM and HO90 mM. All conditions were statistically different (p<0.001) from the 100% DMEM control.

### Plasma-membrane permeability (YO-PRO-1 assay)/apoptosis evaluation (Figure 3)

We assessed apoptosis pathway at 24 h and 48 h using PMP assay in our experimental conditions, hyperosmolarity, BAK, and the associations of both.

At 24 h, compared to DMEM, incubation for 24 h in HO, BAK, and combinations induced a PMP increase (p<0.001), except for HO50 mM and BAK10^−4^%, which induced a nonsignificant increase. Combinations of all BAK concentrations with HO solutions were statistically different (p<0.001) from their respective BAK concentrations alone, except for BAK5.10^−4^% combinations, in which no further cytotoxicity could be observed compared to BAK5.10^−4^% alone.

At 48 h, incubations in the same conditions (HO, BAK, and combinations) induced a time-dependent increase in YO-PRO-1 incorporation compared to 24 h. Combinations of BAK3.10^4^% and BAK5.10^−4^% with all HO did not differ from the respective BAK solutions, as cytotoxicity was too high and the stress alone could not be distinguished from its combinations. All conditions differed (p<0.001) from isotonic DMEM except for BAK 10^−4^%, which still induced a nonsignificant increase at 48 h.

### Caspase-3 activation/apoptosis evaluation (Figure 4)

We evaluated the involvement of caspase-3 activation at 24 h to assess the caspase-dependent apoptosis pathway that could be stimulated by our different challenge.

HO90mM, BAK3.10^−4^%, and combination of BAK3.10^−4^% with HO90 mM induced an increase (p<0.01) in caspase 3 activity of 1.99×, 3.17×, and 9.00×, respectively, compared to control.

### Chromatin condensation (Hoechst 33342)/apoptosis evaluation (Figure 5)

A late apoptosis process was evaluated at 24 h and 48 h using Hoechst 33342 to better-characterized apoptosis following different challenges, hyperosmolar, BAK and the association of both conditions.

At 24 h, compared to DMEM, incubations with HO, BAK, and combinations induced an increase (p<0.001) in fluorescence levels except for HO50 mM, BAK10^−4^%, BAK3.10^−4^%, and for combinations of BAK10^−4^% in HO conditions. No combinations of BAK3.10^−4^% and BAK5.10^−4^% with HO50 mM, HO60 mM, and HO90 mM differed.

At 48 h, incubations in the same conditions (HO, BAK, and combinations) induced a further increase in chromatin condensation compared to 24 h, higher in presence of BAK, and in associations with BAK than with HO solutions alone. All conditions differed (p<0.001) from DMEM for HO conditions except with BAK10^−4^%, BAK10^−4^% with HO50 mM, and HO60mM, which induced a nonsignificant increase in chromatin condensation at 48 h. No BAK5.10^−4^% combinations differed from the BAK solution alone because it was already too toxic for further toxicity to be observed at 48 h.

### Oxidative stress

As BAK is known as an inducer of oxidative stress, we wanted to evaluate at 24 h and 48 h the effects of hyperosmolarity alone and its association with BAK on this production.

### Superoxide anion evaluation (hydroethidine assay, Figure 6)

Incubations for 24 h and 48 h induced a time-dependent increase of fluorescence. Compared to control, 24 h and 48 h incubations in HO60 mM, HO90 mM, BAK5.10^−4^%, the combination of any HO with BAK3.10^−4^% or BAK5.10^−4^% induced a superoxide anion increase, BAK3.10^−4^% showing an increase only at 48 h (p<0.001). At 48 h, all HO conditions combined with BAK3.10^−4^% or BAK5.10^−4^% differed from their respective BAK and HO conditions (p<0.001), except for BAK5.10^−4^% combined with HO90 mM, which did not differ from BAK5.10^−4^%.

### ROS production (H2DCF-DA assay, Figure 7)

Incubations for 24 h and 48 h induced a time-dependent fluorescence increase, higher at 48 h except for BAK5.10^−4^% with HO60 mM. At 24 h and 48 h, compared to control, incubation in BAK5.10^−4^% and the combination of any HO with BAK3.10^−4^% and BAK5.10^−4^% induced a superoxide anion increase (p<0.001), except for HO50 mM and HO60 mM associated with BAK3.10^−4^% at 24 h. HO combinations with BAK3.10^−4^% or BAK5.10^−4^% were more cytotoxic (p<0.001) than their respective BAK and HO conditions, except for HO50 mM associated with BAK3.10^−4^% at 24 h where there was no difference from BAK3.10^−4^%. BAK10^−4^% alone or combined with HO did not differ from DMEM.

### Immunofluorescence microscopy/apoptosis immunodetection (Figure 8 and Figure 9)

**Figure 8:**
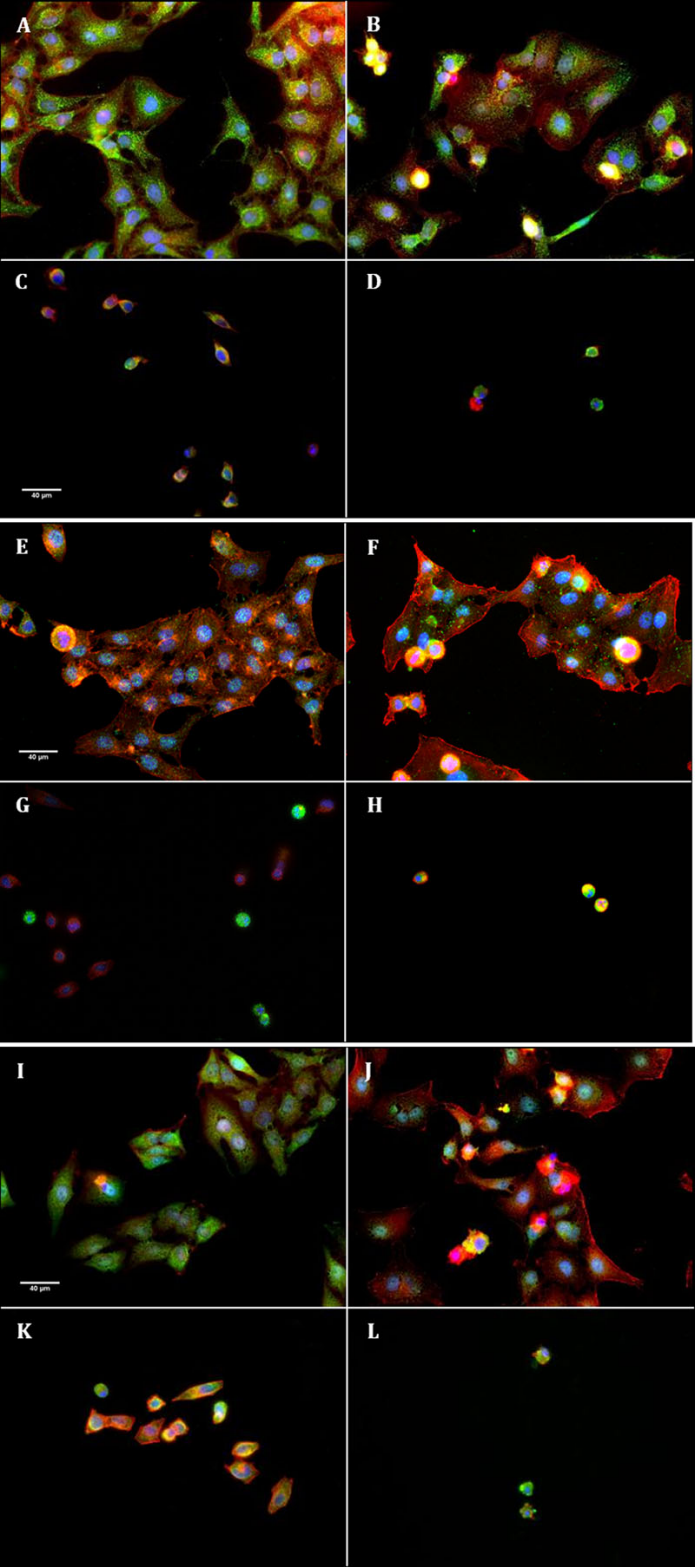
Immunofluorescence of cytochrome c (**A**, **B**, **C**, **D**), active caspase-3 (**E**, **F**, **G**, **H**), and AIF (**I**, **J**, **K**, **L**). Specific stainings appear in green. Nuclear staining was assessed with DAPI (blue) and F-actin cytoskeleton staining with Alexa Fluor-546 phalloidin (red). Cells were stained after 24 h of stress: control cells (**A**, **E**, **I**), HO90 mM (**B**, **F**, **J**), BAK3.10−4% (**C**, **G**, **K**), and the combination of both, HO90 mM+BAK3.10−4% (**D**, **H**, **L**). Cells after HO90 mM stress (**B**) induced a release of cytochrome c from mitochondria (dotted staining) to cytoplasm (diffuse staining) with cytoplasm shrinkage and nuclear condensation after BAK3.10−4% (**C**) and HO90 mM+BAK3.10−4% (**D**) compare to DMEM control (**A**). Note the intense retraction and almost complete destruction of cells submitted to BAK in the two conditions. Increase of caspase-3 fluorescence staining observed in the cytoplasm of HO90 mM-stressed cells (**F**) compared to DMEM (**E**); BAK3.10−4% (**G**) increased caspase-3 in only a few cells; and HO90 mM+BAK3.10−4% (**H**) induced an increase in all stressed cells. AIF was translocated from the mitochondria, appearing as dotted staining for DMEM control (**I**), to the nucleus (nuclear staining) under HO90 mM (**J**), BAK3.10−4% (**K**) and HO90 mM+BAK3.10−4% (**L**).

**Figure 9:**
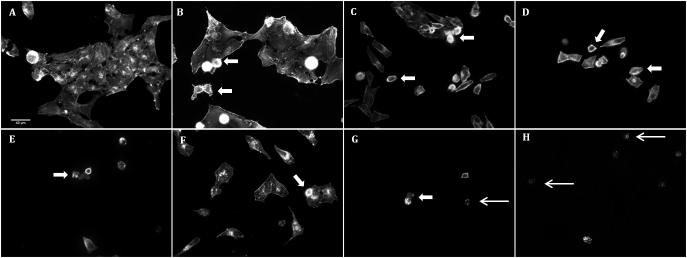
Phalloidin staining (magnification 400×) showing the disorganization of F-actin cytoskeleton in HO90 mM condition (**B**) compared to DMEM control (**A**). BAK10^−4^%-stress (**C**), BAK3.10^−4^% (**D**), and BAK5.10^−4^% (**E**) induced cell blebs, cytoplasm shrinkage, and cell detachment in a dose-dependent manner. All BAK concentrations induced more disorganization and cell shrinkage than even the highest hyperosmolar stress. Combinations of both stresses, namely HO90 mM+BAK10^−4^% (**F**), HO90 mM+BAK3.10^−4^% (**G**), and HO90 mM+BAK5.10^−4^% (**H**) deeply altered cell morphology, such as cytoplasm retraction associated with cell-size decrease (large arrows), and induced a dose-dependent cell death characterized by the presence of apoptotic bodies (thin arrows).

Hyperosmolarity, BAK, and both challenges combined induced caspase-dependent apoptosis. Cytochrome c was released from mitochondria (Figure 8A-D) to the cytoplasm and an increased expression of active caspase-3 was also observed in the cytoplasm of cells stressed by hyperosmolarity, BAK, and combinations (Figure 8E-H). Both stresses also stimulated caspase-independent apoptosis through AIF release from the mitochondrial inner membrane to the nucleus (Figure 8I-L).

Hyperosmolarity induced the disruption of F-actin cytoskeleton, the decrease of several elongated bundles, and the disappearance of a ring-like structure (Figure 9) and altered cell morphology in a time-dependent manner until cell death. These morphological changes were all observed when conjunctival cells were stressed for 24 h with BAK or with combinations of hyperosmolarity with BAK. The F-actin cytoskeleton was severely disrupted, leading to cell detachment and death.

## Discussion

This study simulated various conditions encountered by patients having dry eye by studying the effects of hyperosmotic stress, separately or in combination with BAK, a commonly used eyedrop preservative, to test the hypothesis that BAK administered in an eye already submitted to hyperosmolar conditions would be more toxic than in a healthy, normal ocular surface. We therefore investigated conjunctival cell survival, apoptosis, and oxidative stress in these two conditions because BAK, whose cytotoxicity has been largely demonstrated [33], is still used in many multidose eye drops, such as antiglaucoma treatments, antiallergic agents, or tear substitutes.

We used NaCl to obtain hyperosmolarity ranging from 430 to 500 mOsM, corresponding to high osmolarities compared to normal tear osmolarity (300.8±7.80 mOsM) or even dry eye tear osmolarity (343±32 mOsM) [45,46] to evaluate the cytotoxic effects of osmotic stress. These osmolarities were chosen because our first attempts with lower hyperosmolar media did not reduce cell viability nor did it induce measurable cell changes in our experimental conditions (data not shown). We followed the experiments described by Li et al. [17,18], who had already established that their findings were obtained at an osmolarity of at least 400 mOsM in human corneal or limbal epithelial cells and argued in favor of greater resistance of epithelial cells to hyperosmolar stress in vitro than in vivo [18]. Lower osmolarities in vivo may be sufficient to compromise ocular surface integrity and function because the stress is sustained whereas in vitro higher osmolarities were needed since the stress was only short-term. Moreover, in vitro corneal epithelial cells were shown to adapt to a chronic hypertonic challenge with animal species differences like between rabbit and human corneal epithelial cells. This adaptation occurs through the upregulation of some membrane cotransporter activity involved in the regulatory volume increase in response to hyperosmolar challenges [47].

We tested three BAK concentrations from 10^−4^% to 5.10^−4^%. BAK10^−4^% corresponds to the 1/100 dilution of the 0.01% concentration found in most eyedrops and to a noncytotoxic BAK concentration, as previously reported in similar in vitro models [28,29,48,49]. BAK3.10^−4^% and BAK5.10^−4^% were chosen here to evaluate the impact of cytotoxic BAK concentrations, giving, respectively, 50% and 80% cell death as assessed with the CV assay. As BAK10^−4^% and BAK5.10^−4^% displayed extreme cytotoxic effects from very mild to very strong, respectively, we observed the most relevant findings with the intermediate BAK concentration of 3.10^−4^%.

Conjunctival epithelial cells were sensitive to hyperosmolarity in an osmolarity-dependent manner, but low hyperosmolarity caused limited changes, showing a relative capacity of cells to adapt to hyperosmolar conditions. Under higher hyperosmolar stress or associated toxic factors such as BAK, cells degenerated through an apoptotic mechanism associated with oxidative stress. Hyperosmolarity also induced a time- and dose-dependent increase of PMP. Interestingly, we observed additive cytotoxic effects of BAK and HO. For example, when added to cells submitted to HO90 mM, the lowest BAK concentration became highly cytotoxic. The intermediate concentration, BAK3.10^−4^%, exhibited increased toxic effects with all hyperosmolar solutions, compared to a normal osmolarity condition, showing hyperosmolarity-dependent cytotoxicity.

BAK is known as an inducer of oxidative stress and hyperosmolarity was also shown in some in vitro [50] and in vivo [51] models to induce ROS; we wanted to investigate the effects of the association of both stresses on the ROS production. Oxidative stress was induced by the two experimental stress conditions. Hyperosmolar conditions moderately increased superoxide anion production in an osmotic-dependent manner. This production appeared efficiently compensated as no further ROS production was detected by the H2DCFDA assay showing preserved cell detoxification capacities at these levels of cytotoxicity through detoxifying enzymes such as superoxide dismutase, catalase, or glutathione-peroxidase. BAK is known to impair such protective mechanisms [28], and these data confirmed that it induced a time- and dose-dependent increase in superoxide anion and ROS detection. When the two stresses were combined, BAK seemed to greatly impact the oxidative responses and hyperosmolarity.

We also confirmed the involvement of two major apoptotic pathways, a caspase-dependent one, characterized by cytochrome c release from the mitochondria to cytoplasm and presence of the active caspase-3 and a caspase-independent one through the translocation of AIF from the mitochondria to the nucleus where AIF binds to DNA and leads to chromatin condensation and cell death [52,53]. Here, apoptosis induced by hyperosmolarity was found to have comparable mechanisms to that induced by BAK, which has largely been shown to be proapoptotic on conjunctival cells [28,29,43,48,49]. These two activation pathways can be executed independently or in parallel. Both were involved in hyperosmotic stress, BAK toxicity, and hyperosmolarity associated with BAK. Furthermore, cell detachment, F-actin disorganization, chromatin condensation, and abnormal membrane permeability were all found following apoptosis after hyperosmolar or BAK stresses in our study.

These results support the hypothesis and the frequent clinical observation that BAK, as a preservative contained in many eyedrops, when administered to a patient having dry eye, could dramatically affect conjunctival cells and maintain or aggravate ocular surface impairment. This model is a simple, reproducible in vitro model of NaCl-induced hyperosmolarity on a widely used conjunctival cell line, as already used in similar corneal models [16,17,19,22]. We acknowledge the limitation that this in vitro model cannot fully reflect what occurs in patients, as it is based on only one cell type, exempt of tear film, blood vessels, goblet cells, immune cells, or air flow that interact in complex multifactorial mechanisms [54-58]. To make in vitro culture system closer to in vivo ocular surface dry eye disease, some devices were developed that allow cell exposure to a controlled air flow [58]. Further comparative experimentation with such models would be interesting in future studies. Nevertheless, the conjunctival epithelium remains useful for studying the ocular surface, as it was shown to react to dry eye conditions and cytotoxic stress via expression of HLA DR [59] or secretion of proinflammatory mediators, such as adhesion molecules and chemokines, and to actively participate in disease pathophysiology [60-62].

In conclusion, both hyperosmolarity and BAK induce apoptosis via similar mechanisms, involving caspase-dependent and -independent pathways but with a much higher resistance to hyperosmolarity than to toxic stress. The noteworthy findings of this study are that the combination of a noncytotoxic challenge (hyperosmolarity) and a subtoxic stress (low preservative concentration) can induce substantial cell alterations and cell death, much greater than after each separated stress. This is important from a toxicology point of view, and this model could be valuable for testing the effects of many other non- or minimally cytotoxic compounds such as environmental pollutants or xenobiotics, alone or in combination with a condition mimicking dry eye. Concerning ocular surface diseases, this study highlights the importance of avoiding preservatives like BAK even at low concentrations in a dry eye patient because cytotoxic effects of BAK will act synergistically with hyperosmolarity.

More broadly, the extensive use of BAK over the long-term, as in glaucoma, may progressively cause tear film instability that will enhance hyperosmolarity-induced responses. They were recently reported to increase in several BAK-containing medications [39]. Hence, BAK may cause a condition that will make the ocular surface more sensitive to the toxic compound itself, initiating an exponential toxicity. This could explain the high prevalence of dry eye observed in glaucomatous patients, with a clear relationship between dry eye symptoms and signs, the number of medications and duration of treatments [35,37,38]. In dry eye, tear substitutes preserved with BAK may thus induce a vicious cycle, as BAK may aggravate the consequences of hyperosmotic stress, enhancing ocular surface impairment, and further requiring additional tear substitution. Our in vitro model emphasizes the importance of avoiding BAK in dry eye conditions and for all chronic uses, especially in high-risk patients, those with impaired tear film and ocular surface.

## Supplementary Material

Supporting Movie

## Supplementary Material

Supporting Movie

## Supplementary Material

Supporting Movie
